# Ascites does not accompany pleural effusion developing under dasatinib therapy in patients with CML-CP

**DOI:** 10.1515/pp-2023-0016

**Published:** 2024-02-28

**Authors:** Selin Küçükyurt, Tuğçe Eşkazan, Mesut Ayer, Burçak Kılıçkıran Avcı, İbrahim Hatemi, Ahmet Emre Eşkazan

**Affiliations:** Division of Hematology, Department of Internal Medicine, Cerrahpaşa Faculty of Medicine, Istanbul University-Cerrahpaşa, Istanbul, Türkiye; Division of Gastroenterology, Department of Internal Medicine, Cerrahpaşa Faculty of Medicine, Istanbul University-Cerrahpaşa, Istanbul, Türkiye; Department of Hematology, Başakşehir Çam and Sakura City Hospital, University of Health Sciences, Istanbul, Türkiye; Department of Cardiology, Cerrahpaşa Faculty of Medicine, Istanbul University-Cerrahpaşa, Istanbul, Türkiye

**Keywords:** ascites, chronic myeloid leukemia, chronic-phase chronic myeloid leukemia (CML-CP), dasatinib, pleural effusion

## Abstract

**Objectives:**

Pleural effusion (PE) is the most frequent pulmonary complication of dasatinib, a tyrosine kinase inhibitor (TKI). Concurrent pericardial effusions have been reported in about one-third of the cases. In this study, we aimed to investigate ascites generation in chronic-phase chronic myeloid leukemia (CML-CP) patients developing PE under dasatinib.

**Methods:**

We conducted a cross-sectional study to evaluate whether pericardial effusion and ascites accompany PE in CML-CP patients treated with dasatinib. For this purpose, consecutive patients with CML-CP who developed PE under dasatinib therapy have been evaluated with chest X-ray, transthoracic echocardiography, and abdominal ultrasonography.

**Results:**

There were seven patients, and the median age was 50 years (range, 31–73 years). Most of patients were male (n=5). All patients received imatinib as first-line TKI. Six patients received dasatinib following imatinib failure in second line. The median duration from dasatinib initiation to PE generation was 58 months (range, 8–135 months). Consequently, four patients had grade 1 pericardial effusion, and no patient had ascites.

**Conclusions:**

In our small study, dasatinib-related PE was associated with low-grade pericardial effusion but no ascites. There are hypothetical explanations of this phenomenon including the simultaneous activation/inhibition of kinases; however, more research needs to be performed on this topic.

## Introduction

Dasatinib is a second-generation tyrosine kinase inhibitor (2GTKI), which is utilized in patients with chronic myeloid leukemia in chronic phase (CML-CP), both in the first-line and salvage settings [1, 2]. Other than *BCR::ABL1*, dasatinib also inhibits Src family kinases, *KIT*, and platelet-derived growth factor receptor (*PDGFR*), which is thought to be associated with the nonhematological adverse events (AEs) of this drug [1].

One of the most common nonhematological AEs of dasatinib is pleuropulmonary toxicity. Pleural effusion (PE) can be observed during dasatinib treatment with [3, 4], and among other TKIs, PE is primarily associated with dasatinib [1, 4, 5].

The mechanism underlying PE development is unclear; however, it is thought to be due to an immune-mediated mechanism. It has been suggested that dasatinib provokes serositis (PE and/or pericardial effusion) by causing inhibition of PDGFR beta (PDGFRβ) or Src family kinases, leading to decrease in interstitial fluid pressure or increase endothelial permeability, respectively [3, 6], [7], [8]. Dasatinib-related PEs are generally exudative by the light criteria [7]. Concurrent pericardial effusions have been reported in up to 29 % of the cases [6]; however, to date, no study has been performed evaluating ascites development in patients with CML-CP experiencing PE under dasatinib.

## Materials and methods

We conducted a cross-sectional study to evaluate whether pericardial effusion and ascites accompany PE in patients with CML-CP treated with dasatinib. The primary endpoints of our study were presence of pericardial effusion and ascites. Left ventricular ejection fraction (LVEF), pulmonary artery systolic pressure (PASP), and serum pro-brain natriuretic peptide (pro-BNP) were also measured for the differential diagnosis and etiology of PE. Consecutive patients who were evaluated for dasatinib-related PE between June 2022 and December 2022 were included. PE was detected on physical examination or by imaging (chest X-ray and computed tomography scan if clinically needed). Toxicities were graded using the U.S. National Cancer Institute’s Common Terminology Criteria for Adverse Events (CTCAE) [9]. Serum pro-BNP was tested in all patients, and transthoracic echocardiography (TTE) (Philips Medical Systems, Bothell, Washington, USA) with an S5-1 probe was performed by a cardiologist (BKA), and LVEF and PASP were measured. In addition, abdominal ultrasonography (AUS) (Hitachi HI VISION Avius - Diagnostic Ultrasound System, Japan) was performed by two gastroenterologists (TE or İH) to evaluate ascites. TTE and AUS were performed once according to the study protocol. If effusion was detected, follow-up imaging was planned to be continued intermittently. This study was approved by the Ethics Committee on Non-Interventional Studies (30.06.2022-415462).

## Results

A total of seven patients were included (Table 1). The median age was 50 years (range, 31–73 years). Five patients were male, and all cases were in first CP. Six patients (83.3 %) had at least one comorbidity. All patients received imatinib as first-line TKI, and according to the Sokal score, four patients were low-risk. Six patients received dasatinib following imatinib failure in second line, and dasatinib was administered as a fourth-line therapy in Patient #5. The median duration from diagnosis to dasatinib initiation was 27 months (range, 14–86 months). The reasons for switching to dasatinib were suboptimal response in four patients, loss of major molecular response (MMR) in two, and grade 3 bosutinib-related diarrhea in one (Patient #5) (Table 1).

**Table 1:** Baseline characteristics, clinical and laboratory features of patients experiencing pleural effusion under dasatinib.

Patient #	Sex	Age at diagnosis, years	Comorbidities	Sokal risk score	Duration from diagnosis to dasatinib initiation, months	Reason for switching to dasatinib	Time from dasatinib initiation to PE generation, months	Dasatinib dose at the time of PE detection	PE grade	Presence of pericardial effusion (yes/no)	Pro-BNP, ng/dL	TTE findings	Ascites by AUS
1	M	50	HT, AF, CAD, HL, PVD	High	35	Suboptimal response	32	100 mg QD	2	Yes	491	PASP: 34 mmHg	Absent
												LVEF: 55 %	
2	F	55	Asthma	Low	16.5	Suboptimal response	63	100 mg QD	2	Yes	42	PASP: 38 mmHg	Absent
												LVEF: 60 %	
3	F	60	DM, HT, HL, CAD, CHF, CKD, VHD	Low	29	Suboptimal response	135	50 mg QD	2	Yes	1194	PASP: 49 mmHg	Absent
												LVEF: 53 %	
4	M	31	None	Low	19.5	Loss of MMR	39	100 mg QD	2	Yes	10	PSAP: 30 mmHg	Absent
												LVEF: 60 %	
5	M	73	HT, CAD, arrhythmia	Intermediate	27	Grade 3 bosutinib-related diarrhea	8	50 mg QD	3	No	5126	PSAP: 50 mmHg	Absent
												LVEF: 55 %	
6	M	31	HT, OSA	Low	86	Loss of MMR	89	100 mg QD	2	No	10	PASP: 32 mmHg	Absent
												LVEF: 55 %	
7	M	46	DM, HL	Intermediate	14	Suboptimal response	58	100 mg QD	2	No	236	PASP: 25 mmHg	Absent
												LVEF: 49 %	

AF, atrial fibrillation; AUS, abdominal ultrasonography; BNP, brain-natriuretic peptic; CAD, coronary artery disease; CHF, coronary heart failure; CKD, chronic kidney disease; DM, diabetes mellitus; F, female; HL, hyperlipidemia; HT, hypertension; LVEF, left ventricular ejection fraction; M, male; MMR, major molecular response; OSA, obstructive sleep apnea; QD, once daily; PASP, pulmonary artery systolic pressure; PE, pleural effusion; PVD, peripheral venous disease; TTE, transthoracic echocardiography; VHD, valvular heart disease.

The median duration from dasatinib initiation to PE generation was 58 months (range, 8–135 months). The starting dose of dasatinib was 100 mg once daily (QD) in five patients, and Patient #5 started dasatinib at 50 mg QD due to advanced age, comorbidity, and poor performance status. In addition, dasatinib dose was reduced to 50 mg QD in Patient #3 due to grade 2 PE occurring at the 49th month of dasatinib.

Six of seven patients had grade 2 PE, and Patient #5 developed grade 3 PE requiring tube thoracostomy. In five patients, PE regressed after short-term furosemide and corticosteroids, together with temporary discontinuation of dasatinib; however, nilotinib was switched in Patient #2 due to possible dasatinib-induced pulmonary arterial hypertension (PAH).

**Figure 1:**
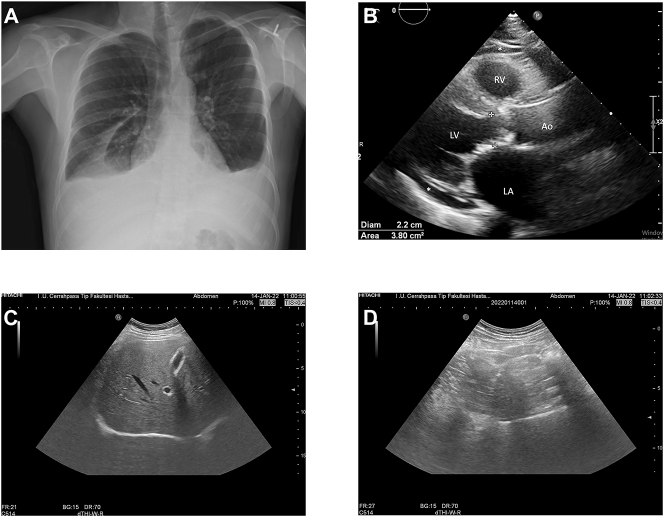
The chest X-ray, transthoracic echocardiography, and abdominal ultrasonography images of Patient #4. (A) Bilateral pleural effusion in chest X-ray. (B) Minimal pericardial effusion detected in TTE (Ao, aorta; LA, left atrium; LV, left ventricle; RV, right ventricle. *Parasternal long-axis view demonstrating pericardial effusion). (C and D) AUS showing no ascites in the perihepatic area (C) and no peritoneal fluid between the bowel loops (D).

At the time of PE detection, LVEF was >50 % in six patients by TTE, and serum pro-BNP levels were high in four patients, of which two (Patients #2 and #3) had findings suggestive of PAH in TTE (Table 1). Moreover, mean PASP was 34 mmHg (range, 25–50 mmHg) measured by TTE. Patient #2 didn’t undergo right heart catheterization (RHC), and dasatinib was permanently stopped. Patient #3 had an RHC, which did not confirm PAH diagnosis. Four patients had grade 1 pericardial effusion accompanying PE by TTE. Ascites was not detected in any of our patients by AUS. The chest radiography, TTE, and AUS images of Patient #4 were shown in Figure 1.

## Discussion

Dasatinib is a second-generation TKI approved in the treatment of CML. PE is one of the most common nonhematological adverse events of dasatinib. In our study, our aim was to investigate pericardial effusion and ascites generation in patients with CML-CP who developed PE during dasatinib therapy. After all, 57 % (4/7) of the cases had pericardial effusion, but we didn’t detect ascites in any patient.

Dasatinib-related fluid retention is a well-known side effect. Case series of dasatinib-related pericardial effusion accompanied by pleural effusion have been previously reported [6, 10]. However, ascites generation in CML patients receiving dasatinib has not been evaluated previously. Although many conditions may cause ascites, the most common cause is liver cirrhosis [11]. There is one case report published in the literature, presenting a CML patient experiencing ascites accompanying dasatinib-related bilateral PE; however, this patient had concomitant alcoholic liver disease [12]. He had *exudative PE*, on the other hand, ascitic fluid was transudate. Most probably, the ascites in this particular case was not due to dasatinib use depending on both the nature of the ascitic fluid and the underlying liver pathology.

The mechanism underlying PE development is unclear. There is a view that pleural manifestations may occur during dasatinib treatment, possibly related to an immune-mediated mechanism rather than fluid retention. The reason for this, in previous publications, PE under dasatinib was thought to be related to factors such as lymphocyte infiltration in the pleural fluid, the presence of autoantibodies and concomitant autoimmune diseases, and steroid responsiveness of the effusion [3], [4], [5], [6], [7], [8]. Serosal involvement can frequently be seen in systemic inflammatory diseases such as systemic lupus erythematosus and rheumatoid arthritis and not all serosal surfaces have to be affected at the same time. Also, serosal involvement usually reflects the activity of the systemic disease [3], [4], [5], [6], [7], [8]. In our case series, no immune-mediated mechanism mentioned above was noted. Presumably, the occurrence of fluid retention is multifactorial.

Mechanism of dasatinib-related PE development was investigated previously in an animal model [13]. Rats were treated with daily imatinib, dasatinib, or vehicle [dimethylsulfoxide (DMSO):saline (1:3, v/v)] intraperitoneally for 8 weeks. Imatinib and dasatinib were given at three and two different doses, respectively. None of the imatinib doses, low-dose dasatinib, and vehicle-induced PE. On the other hand, seven of the eight rats receiving a high dose (10× clinically relevant dose) of dasatinib had PE by 8 weeks. No pericardial effusion or ascites was detected in rats receiving imatinib, dasatinib, or vehicle. In this study, it was shown *in vivo* and *in vitro* (human umbilical vein endothelial cells) that dasatinib dose-dependently and reversibly increases pulmonary endothelial permeability and causes PE with a ROS-dependent mechanism. The lung-specific effects of dasatinib may be related to the high hydrostatic gradient in the lung and pleural capillaries. Whereas interstitial pressure is low in the pericardium and abdominal cavity [13]. As dasatinib may induce pericardial effusion in humans, low interstitial pressure alone may not be the explanation for this condition.

It has been postulated that PDGFRβ plays a role in the pathogenesis of PE by causing increased endothelial permeability. PDGF is a dimeric molecule, so it can bind two receptors simultaneously, forming a bridge between the receptors. Some of the cells expressing PDGFRβ receptors include fibroblasts, vascular smooth muscle cells, and capillary endothelial cells. Itoh cells of the liver also express PDGFRβ; however, liver sinusoidal endothelial cells do not [14]. If only PDGFR had a role in the development of dasatinib-related PE, PDGFRβ inhibition would have caused increased capillary permeability, leading to ascites due to the changes in interstitial pressure [14]. Supporting this, PDGFRβ blocking agent (CDP-860) had been shown to cause ascites and/or PE in patients with advanced ovarian and colorectal cancer, which was not a disease-specific phenomenon [15]. The simultaneous inhibition of other kinases that counteract this biological effect may play a role in the development of dasatinib-related PE and the absence of ascites.

In conclusion, PE under dasatinib is a well-known toxicity of the drug. Dasatinib may also cause pericardial effusion, but development of *de novo* ascites during dasatinib therapy has not been investigated previously in patients with CML-CP. Although the number of patients in our case series is relatively low, we, to the best of our knowledge, have shown for the first time that ascites does not accompany PE developing under dasatinib. There are hypothetical explanations of this phenomenon including the simultaneous activation/inhibition of kinases; however, more research needs to be performed on this topic. Other serosal manifestations should be evaluated in patients with PE detected in subsequent investigations. If possible, fluid sampling could be performed and both inflammatory cytokines and growth factors could be measured to address the underlying mechanism of this *off-target* effect of dasatinib.
